# Assessing One Health capacities for transboundary zoonotic diseases at the Libya-Tunisia border

**DOI:** 10.1186/s42522-024-00101-z

**Published:** 2024-03-19

**Authors:** Lauren N. Miller, Walid K. Saadawi, Wafa Ben Hamouda, Ahmed S. Elgari, Emaduldin A. Abdulkarim, Ashur M. M. Lmrabet, Abir E. Elbukhmari, Kaouther Harabech, Ammar Ali Jemai, Milad Farhat, Rasha Al-Azab, Abdulaziz Zorgani, Omar Elamher, Tarek Al Sanouri, Claire J. Standley, Erin M. Sorrell

**Affiliations:** 1 Center for Global Health Science and Security, Georgetown University Medical Center, Washington, DC USA; 2 National Centre for Disease Control, Tripoli, Libya; 3 General Directorate for Veterinary Services, Ministry of Agriculture, Water Resources and Fisheries, Tunis, Tunisia; 4 National Centre for Animal Health, Tripoli, Libya; 5 Ministry of Public Health, Tunis, Tunisia; 6 CDRA, Ministry of Agriculture, Water Resources and Fisheries, Tunis, Tunisia; 7 Eastern Mediterranean Public Health Network, Amman, Jordan; 8 Heidelberg Institute of Global Health, University of Heidelberg, Heidelberg, Germany; 9 Center for Health Security, Department of Environmental Health and Engineering, Bloomberg School of Public Health, Johns Hopkins University, Baltimore, MD USA

**Keywords:** One health, Transboundary zoonotic diseases, Disease prioritization tools, Multisectoral engagement, Cross-border collaboration, Points of entry, Ground crossing, Emerging infectious diseases

## Abstract

**Background:**

The dynamic nature of zoonotic emergence, spillover and spread necessitates multisectoral coordination beyond national borders to encompass cross-boundary and regional cooperation. Designated points of entry (POEs), specifically ground crossings, serve as critical locales for establishing and maintaining robust prevention, detection, notification, coordination, and response mechanisms to transboundary emerging and re-emerging disease threats. In order to better assess One Health capacities for transboundary zoonotic diseases (TZD) prevention, detection and response we adapted an existing tool, One Health Systems Assessment for Priority Zoonoses (OHSAPZ), for a cross-border, POE setting in North Africa.

**Methods:**

The One Health Transboundary Assessment for Priority Zoonoses (OHTAPZ) tool was used to support prioritization of transboundary zoonoses and analyze operational capacities between national and subnational-level human and animal health stakeholders from Libya and Tunisia. Country partners jointly identified and prioritized five TZDs of concern. Case study scenarios for each priority pathogen were used to elicit current disease operations, as well as multisectoral and bilateral engagement networks. Finally, a gap analysis was performed to determine bilateral strengths and weaknesses to TZDs.

**Results:**

The five priority TZDs jointly confirmed to undergo One Health assessment were avian influenza (low and high pathogenic strains); brucellosis; Rift Valley fever; Crimean-Congo hemorrhagic fever; and rabies. Using the qualitative information collected, a transboundary systems map schematic was developed outlining the movement of human patients, animals, diagnostic samples, and routes of communication and coordination both within and between countries for zoonotic diseases.

**Conclusions:**

Analysis of current operations (prevention, detection, surveillance, laboratory capacity, quarantine/isolation, and response) and the resulting transboundary systems map schematic helped identify existing capacity strengths for certain priority pathogens, as well as challenges to timely information-sharing and coordination. We developed targeted recommendations to address these limitations for joint action planning between Libya and Tunisia.

## Background

In order to support global preparedness, notification and response to disease emergence and re-emergence, nations must take appropriate steps to build capacity for prevention, detection, reporting, and management of zoonotic threats. Zoonoses capitalize on the overlapping complexities between humans, animals, and the evolving climate and environment, challenging national capabilities to cross-coordinate, unify preparedness schemes and activate response and recovery operations towards a One Health approach. Competing health priorities, funding limitations, resource inadequacies and interdisciplinary silos can all impact the effectiveness and sustainability of national and regional One Health strategies [1, 2]. A number of national One Health disease prioritization and assessment tools and resources have been developed and used by national governments, international organizations and other relevant stakeholders to support interdisciplinary approaches to outbreak preparedness and response [3–10]. Nevertheless, the dynamic nature of zoonotic emergence, spillover and spread necessitates multisectoral coordination beyond national borders to encompass cross-boundary and regional cooperation. This is particularly acute with respect to transboundary animal diseases (TADs), which are highly contagious infectious diseases, some capable of reaching epidemic or pandemic levels, that disproportionally impact low- and middle-income countries [11]. While not solely zoonotic in nature, TADs are considered food and agricultural security targets due to their potential for significant socioeconomic and public health consequences [11]. Both the Food and Agriculture Organization of the United Nations (FAO) and World Organisation for Animal Health (WOAH) maintain a list of TADs of international concern [12]. FAO and WOAH’s joint Global Framework for the Progressive Control of Transboundary Animal Diseases (GF-TADs) supports the creation of sub-regional, regional and global networks for prevention, detection, and control of priority TADs, and its subsequent five-year strategy helps construct operational plans and monitoring tools to track progress [13, 14]. While these global initiatives concentrate on TADs, to date there has been less emphasis on transboundary zoonotic diseases (TZDs), which have similar public health challenges and devastating socioeconomic impacts if not effectively detected and properly contained [15]. TZDs, by their nature, also require multisectoral coordination and capacity building for prevention, detection and response both at a national level and through multilateral coordination at points of entry (POE).

Infectious diseases are spread through the interaction and movement of travelers, animals, vectors and goods. This movement occurs both at formal and informal POEs, as demonstrated by severe acute respiratory syndrome (SARS), Ebola virus disease, and most recently coronavirus disease 2019 (COVID-19) [16]. Emerging infectious diseases, particularly TZDs, can be driven by diverse ecological, political, and socioeconomic factors, and the nature of their transboundary spread can be both positively and negatively influenced by sectors’ capacities and involvement [17]. For nations experiencing conflict and civil unrest, governments are further challenged to develop surveillance systems and implement consistent TZD control measures, such as enforced quarantine, including at POEs, due to institutional breakdowns and physical inaccessibility [18].

In order to better assess One Health capacities for TZD prevention, detection and response we adapted an existing tool for a cross-border, POE setting in North Africa. The One Health Systems Assessment for Priority Zoonoses (OHSAPZ) tool utilizes a phased methodology to map existing nodes of communication and coordination between multisectoral stakeholders for prevention, detection, response and recovery to national priority zoonoses [7, 15]. Since 2015, this assessment methodology has been applied in a variety of country settings spanning from the Middle East to North and West Africa, successfully adapted to accommodate in-country contexts, and completed wholly remotely using facilitated videoconference technologies [8–10]. In collaboration with One Health stakeholders in Libya and Tunisia, we adapted the OHSAPZ methodology to a transboundary setting, focusing the approach on existing multisectoral and bilateral One Health capacities and coordination for priority TZDs at POEs.

## Methods

The development of the One Health Transboundary Assessment for Priority Zoonoses (OHTAPZ) derived from the well-tested OHSAPZ methodology and accompanying papers [7–10, 15]. As such, the previously established three-phased approach of prioritization, systems mapping, and analysis and recommendations was adapted and applied to a transboundary context. This section briefly describes how these phases were implemented with respect to transboundary assessment. This work began in the summer of 2022 and was completed in the spring of 2023. Please refer to the 2023 OHSAPZ manual (3rd edition) for the complete methodology [7].

### Phase 1: Prioritization

The major differences in the four prioritization steps between OHSAPZ and OHTAPZ methodologies are comparisons in the distribution, prevalence, and burden of national and transboundary zoonoses, an expansion in stakeholder mapping, and the selection criteria used to consolidate a joint list of priority TZDs. To assess transboundary zoonotic risks we reviewed reporting through Programing for Monitoring Emerging Diseases (ProMed) online national surveillance reports, regional reporting networks, and outbreak notifications to WOAH’s World Animal Health Information System (WAHIS) [19, 20]. Our list for prioritization included both nationally reported zoonoses as well as possible transboundary threats.

Our stakeholder mapping considered both traditional One Health ministries and expanded to sectors responsible for border health. This included sector-specific partners leading customs and immigration health screening for individuals, animals and animal products (meat, eggs, etc.) that enter and exit through formal ground crossings. We developed key questions to validate the various stakeholders within the human, animal and border control sectors and determine their roles and capacities in TZD detection, surveillance, reporting, response and/or cross-coordination or collaboration for TZDs at ground crossings.

The primary objective of the prioritization phase was to create a consensus list of approximately five TZDs for consideration. To reach consensus on a bilateral list of priority TZDs to undergo assessment, stakeholders first ranked national priority TZDs. An expanded list of selection criteria was applied including considerations for WOAH/FAO’s TADs list, existing mechanisms for cross-border stakeholder communication and coordination, and disease acceleration by animal/human movement. Once national TZDs were selected, country partners were then asked to consolidate their national lists into a joint, bilateral priority list of five TZDs.

### Phase 2: Transboundary systems mapping

The objective of phase two was to use the priority TZDs as case studies to map existing processes for communication and coordination. The three steps under phase 2 were minimally altered for the transboundary methodology. Case study scenarios were developed for each priority TZD with a particular focus on events occurring or involving formal land border crossings. These scenarios were used to collect information related to seven major competencies: prevention; surveillance; detection; laboratory capacity; quarantine/isolation; response; and communication and coordination. National and transboundary responses were compared and the information captured was used to outline reporting processes, from case identification at ground crossings up to national-level authorities. Networks currently in place for rapid detection, notification, and response to cases were mapped and used to identify current intersectoral and transboundary communication and coordination mechanisms. Our ultimate products were a disease-agnostic systems map schematic and accompanying detailed narrative that outlined national and transboundary operations. The disease-agnostic map schematic captures the systems available for the designated priority TZDs and highlights strengths and gaps for future targeted, capacity-sustaining and capacity-building resources.

### Phase 3: Analysis and recommendations

The objective of phase three was to use the systems maps to analyze the strengths and weaknesses of existing coordination and develop actions to address any gaps identified. The analysis and recommendations steps mirrored the OHSAPZ process with only minor changes to incorporate the additional sectors involved.

## Results

### POEs in Libya and Tunisia

A total of 25 formal POEs are located across Libya with 3 designated seaports and 3 ground crossings under the International Health Regulations (IHR) [21, 22]. Of the 26 formal POEs in Tunisia, there are 8 IHR-designated airports, 8 authorized seaports, and 2 designated ground crossings [23, 24]. There are two official ground crossings located between Libya and Tunisia: Ras Ajdir (Jedir or Jdir) in the north and Dehiba Wazin in the south. At the time of this pilot, there was no joint IHR-designated ground crossing between Libya and Tunisia.

### Key Transboundary Stakeholders and Capacities

**Fig. 1:**
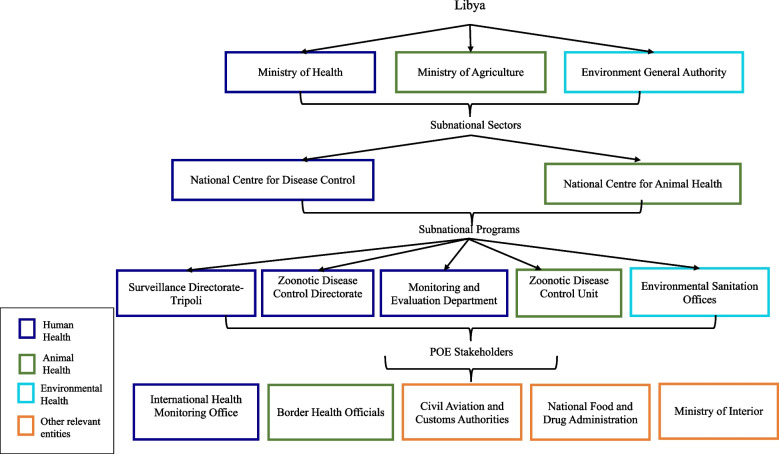
Key Multisectoral Stakeholders Identified in Libya and Tunisia. Tiered schematic of relevant ministries, subnational sectors, relevant programs, supporting partners, and POE stakeholders with aligned priorities identified as key stakeholders in prevention, detection, notification and response of transboundary zoonotic diseases in Libya and Tunisia. Human health entities are represented in dark blue; animal health sectors are green; environmental health correspond with light blue; and orange is used to depict other relevant entities

Using the OHTAPZ approach described above, we applied the phased methodology to identify existing networks in place between key border health stakeholders within and across Libya and Tunisia’s two official land crossings. Stakeholders across public, veterinary and border health in Libya and Tunisia were identified and validated using the OHTAPZ criteria. Stakeholders included national ministries, subnational sectors, relevant programs, supporting partners and POE stakeholders (Fig. 1).

For Libya, the National Centre for Disease Control (NCDC) and National Centre for Animal Health (NCAH), both subnational government sectors, were identified as key stakeholders responsible for national prevention and control of human and animal diseases. NCDC’s International Health Monitoring Office (IHMO) is responsible for all public health operations at POEs including monitoring international outbreaks, managing and implementing public health operations, and health checks at immigration. With approximately 200 personnel on staff, these border health officials routinely communicate with officials at headquarters in Tripoli. The IHMO shares information with the NCDC’s Zoonotic Disease Control Directorate which leads all TZD operations and the Surveillance Directorate which collects and analyzes any data reported from POEs. Within the Ministry of Agriculture, NCAH oversees all veterinary services and animal health in Libya, including examinations for imported and exported animals and implementation of quarantine measures. The NCAH staffs approximately 120 border health officials at POEs and designated quarantine locations. The NCAH also has a Zoonotic Disease Control Unit responsible for TZDs in Tripoli.

In Tunisia, the Ministry of Public Health (MoPH) and the Ministry of Agriculture, Water Resources and Fisheries/General Directorate for Veterinary Services (MoA/VS) were identified as key stakeholders. The MoPH is responsible for leading and managing all public health operations, including at POEs. The number of staffing is dependent on the size and traffic experienced by a POE; for ground crossings, MoPH provides at least one public health agent on 24 h-duty. The VS is the national agency within the MoA responsible for monitoring and controlling animal diseases and the products of animal origin in Tunisia. MoA/VS staffs border officials at POEs to certify import and export documentation and communicate concerns to the national level. A total of 260 veterinarians are on staff for POEs, with at least one veterinarian per POE in Tunisia.

### Selection criteria and TZD prioritization

The disease prioritization process resulted in a list of five national priority TZDs for Libya and Tunisia, using 16 of the 22 available selection criteria (Table 1).

**Table 1:** TZD Selection Criteria and Findings from Libya and Tunisia’s Rated Scale of Importance

Selection Criteria	Rank Score (0–2)		
	**Libya**	**Tunisia**	**Final Selection Criteria**
Endemic in country or region (known history of transboundary spread)	2	2	**Yes**
Epidemic potential in country or region	2	2	**Yes**
Potential for endemic or pandemic in humans or animals	2	2	**Yes**
Pathogen of international concern – reportable to World Health Organization (WHO)	2	2	**Yes**
Large disease burden in humans (morbidity and/or mortality)	2	2	**Yes**
Large disease burden in livestock or domestic animals (morbidity and/or mortality)	2	2	**Yes**
Large impact on imports/exports	2	2	**Yes**
Regional priority disease	2	2	**Yes**
Economic, environmental or social impact	2	2	**Yes**
Bioterrorism potential	2	2	**Yes**
Mode of transmission	2	2	**Yes**
Accelerated by animal/human movement (legal and/or illegal)	2	2	**Yes**
Emerging potential in country or region	2	1	Yes*
Pathogen of international concern – reportable to WOAH (OIE)	2	1	Yes*
Listed on national notifiable disease list (MOH or MOA)	2	1	Yes*
Listed on WOAH or FAO Transboundary Animal Disease List	2	1	Yes*
Large disease burden in wildlife (morbidity and/or mortality)	1	1	No
Available and accessible treatments (vaccines, countermeasures)	1	1	No
Existing mechanisms for multisectoral stakeholder communication and coordination	1	1	No
Available control strategies/programs	1	1	No
Available laboratory diagnostics (central and sub-national level)	1	0	No
Existing mechanisms for cross-border stakeholder communication and coordination	0	1	No

A comprehensive list of selection criteria was used by key stakeholders to narrow down priority transboundary zoonotic diseases (TZDs) to undergo One Health assessment. The table summarizes Libya and Tunisia’s scaled rating of criteria which informed the final list of criteria for bilateral TZD prioritization. Criteria were ranked as 2 (most important), 1 (moderately important) or 0 (not important) by each country group. Yes = consensus to include; Yes* = no consensus but moves forward as one country designated a score of 2; No = score < 2 from both countries, criterion is eliminated from final consideration.

Tunisia identified highly pathogenic avian influenza (HPAI), brucellosis, Rift Valley fever (RVF), rabies, and West Nile virus as their national TZDs while Libya selected avian influenza (low and high pathogenic strains), brucellosis, RVF, rabies and rickettsiosis as national TZDs. Consensus regarding the final list was reached without any major disagreements among stakeholders. The final bilateral TZD list consisted of avian influenza (low and high pathogenic strains); brucellosis, mosquito-borne diseases; tick-borne diseases; and rabies (Table 2). Both countries agreed that mapping vector-borne diseases by arthropod vector would be the best way to capture existing capacities and selected RVF to represent mosquito-borne diseases and Crimean-Congo hemorrhagic fever (CCHF) to represent tick-borne diseases in the specific case study discussions. Summary information on the epidemiology and occurrence of these priority TZDs in Libya and Tunisia, as determined through our literature review, is provided in Table 3.

**Table 2:** Final qualifying criteria and findings from Libya and Tunisia’s TZD prioritization

Final qualifying criteria	Pathogen									
	**Tunisia**					**Libya**				
	**Rabies**	**Brucellosis**	**RVF**	**HPAI**	WNV	**Rabies**	**Brucellosis**	**RVF**	**HPAI/** **LPAI**	Rickettsia
Endemic in country or region (known history of transboundary spread)	X	X				X	X			X
Epidemic potential in country or region		X	X	X	X			X	X	X
Emerging potential in country or region			X	X	X			X	X	
Potential for endemic or pandemic in humans or animals		X	X	X			X	X	X	X
Pathogen of international concern – reportable to WHO			X	X	X			X	X	
Pathogen of international concern – reportable to WOAH	X	X	X	X	X	X	X	X	X	
Large disease burden in humans (morbidity/ mortality)	X		X		X	X	X	X	X	
Large disease burden in livestock or domestic animals (morbidity/ mortality)		X	X	X			X	X	X	
Large impact on imports/exports		X		X					X	
Listed on national notifiable disease list	X	X	X	X		X	X	X	X	
Listed on WOAH or FAO Transboundary Animal Disease List	X	X	X	X		X	X	X	X	
Regional priority disease	X	X	X	X		X	X	X	X	
Economic, environmental, or social impact	X	X	X	X		X	X	X	X	
Bioterrorism potential		X	X				X	X		
Mode of transmission	X	X		X	X	X	X			X
Accelerated by animal/human movement (legal and illegal)		X	X	X			X	X	X	

The table showcases the final qualifying criteria used by Libyan and Tunisian stakeholders to discuss and select bilateral TZDs from their respective national TZDs. The consensus bilateral TZDs are bolded. *Abbreviations*: *RVF* Rift Valley fever, *HPAI/LPAI* Highly pathogenic avian influenza, low pathogenic avian influenza, *WNV* West Nile virus. An “X” is used to indicate the presence of evidence

**Table 3:** Epidemiology and occurrence of priority TZDs in Libya and Tunisia

Pathogen	Endemic		Epidemic Potential		Emerging/ Re-emerging		Cases Reported (2013–2023)				Transboundary Spillover Risk	
							**Tunisia**		**Libya**			
	**Tunisia**	**Libya**	**Tunisia**	**Libya**	**Tunisia**	**Libya**	**Animal**	**Human**	**Animal**	**Human**	**Tunisia**	**Libya**
Avian Influenza^a,b^			X	X	X	X	X		X	X	X	X
Brucellosis ^b^	X	X					X	X	X	X	X	X
RVF ^a,b^			X	X	X	X			X		X	X
CCHF ^a,b^			X	X	X	X		X			X	X
Rabies ^b^	X	X					X	X	X	X	X	X

Epidemiology, outbreak occurrence in humans and animals over the last ten years, and transboundary spillover risk for the five priority TZDs in Libya and Tunisia. *Abbreviations*: *RVF* Rift Valley fever, *CCHF* Crimean-Congo hemorrhagic fever. An “X” is used to indicate the presence of evidence following literature review

^a^Notifiable to WHO

^b^Notifiable to WOAH

### Transboundary systems map development

**Fig. 2:**
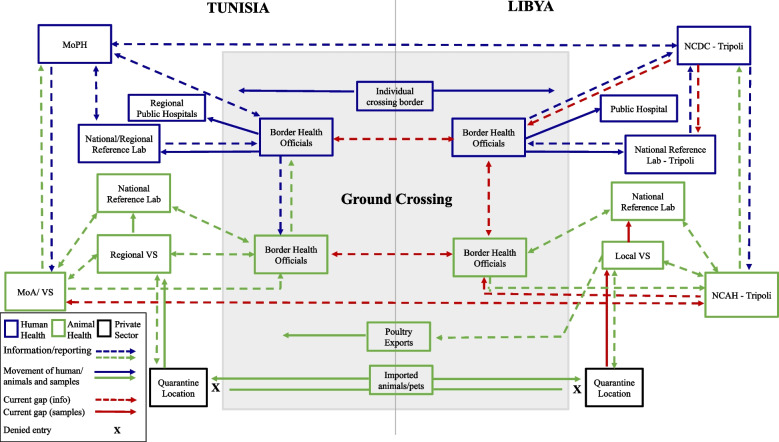
Disease-Agnostic Transboundary Systems Map Schematic at the Tunisia-Libya Border. The figure depicts human and animal movement, sample referral, and information sharing networks for TZDs at formal ground crossing settings between Tunisia and Libya. Efforts led by human health sectors are represented in dark blue while those led by animal health sectors are in green. Solid arrows represent movement of humans, animals and/or diagnostic samples. Arrows with dashes represent surveillance data and information sharing/reporting. Formal capacity, for at least one or more of the priority TZDs, was captured as a strength. Major disease-specific discrepancies are captured in the narrative. Red arrows (dashed denote data/reporting, solid denote patient/sample referral) indicate a lack of formal capacity or current gaps. Informal chains of communication or mechanisms that exist only in emergency settings were designated as gaps. Black boxes around quarantine centers indicate operation by a private entity. Abbreviations: NCDC = Libyan National Centre for Disease Control; NCAH = Libyan National Centre for Animal Health; MoPH = Tunisian Ministry of Public Health; MoA/VS = Tunisian Ministry of Agriculture/Veterinary Services; VS = Veterinary Services

Using the data collected from our case study scenario discussions on the five consensus TZDs, a disease agnostic transboundary system map schematic was created, outlining the routes of communication and coordination that support the tracking and/or treatment/quarantine/isolation of human patients and animals, and control strategies for the priority zoonoses (HPAI, brucellosis, rabies) at formal land border crossings between Libya and Tunisia (Fig. 2). This information was also captured in narrative form below and outlined in the seven capacity categories used in our case study scenarios.

## Findings

### Prevention

Border health officials are located in the same offices at POEs in Tunisia, providing a supportive operating environment for public and veterinary health to coordinate and collaborate. MoPH has protocols for vector monitoring and surveillance at POEs. Routine surveillance is conducted on a monthly basis while some high-risk locations experience additional surveillance activities. Routine entomological surveys are conducted at all POEs, including ground crossings on the border with Libya, and findings are shared with public health counterparts when mosquito species of interest are detected. Currently, only yellow fever and malaria risk communications are available for travelers. National awareness and educational campaigns are routinely implemented by MoPH for rabies and brucellosis but do not extend to POEs. While MoA/VS has a national strategy and control program for brucellosis (MoPH’s strategy is under development) there is a recognized need to engage neighboring countries towards the creation of an appropriate transboundary strategy. To prevent TZD spread into Tunisia, border officials routinely monitor a list of countries authorized by MoA for animal import; the list is updated daily based on regional and international surveillance reports. Transboundary disease resources and trainings are available for both human and animal health officials in Tunisia. MoPH border health officials conduct special activities for pandemic and emergency preparedness including contingency planning for airports and seaports, training on IHR-mandated routine and emergency capacities for POEs, and risk assessments for reporting to the IHR national focal point (NFP). MoA/VS has even implemented an HPAI simulation for farmers and importers however border officials lack specialized training on specific TAD and TZD detection and specific TZD outbreak simulation trainings at POEs.

In Libya, while inconsistently implemented, vector and stray dog control are the responsibility of municipal Environmental Sanitation Offices (ESOs) at the national level while NCDC is responsible for vector surveillance, monitoring, and control specifically at POEs. A national vector control plan for POEs is currently under development. NCDC and NCAH conduct various public awareness campaigns for most of the priority TZDs across Libya (NCAH does not include brucellosis) however there are no educational materials, infographics, or risk communications made available at POEs. Libya NCDC provides TZD awareness training, with a particular focus on vector-borne diseases, to border health officials. NCAH does not currently provide TZD training opportunities for border health officials.

### Surveillance

Routine communication and reporting of TZD cases can occur directly at Tunisia’s POEs with border health sectors co-located on site. Border health officials have protocols in place for monitoring and surveillance of all five priority TZDs at all POEs and communicate with MoPH and/or MoA/VS as needed. MoPH border health officials conduct entomological monitoring for vector-borne diseases, surveillance of travelers at immigration particularly for brucellosis and influenza. A national Electronic Integrated Disease Surveillance System (EIDSS) and an integrated monitoring system is available for rabies and brucellosis. A network of breeders, private and public veterinarians, and veterinary laboratories are responsible for notifying cases to the MoA/VS. In addition to strict surveillance, the MoA requires VS to perform routine testing for avian influenza in migratory and domestic bird populations, particularly poultry/products sold by street vendors and at markets, to help maintain Tunisia’s annual declaration as an avian influenza-free country. A multisectoral surveillance network can be activated in the event of unusual bird die-offs to initiate sampling and investigation. Animal importation from countries with any suspected TADS or TZDs is forbidden however the constant threat of migration and illegal trade present a variety of challenges to TZD prevention and surveillance. In response, MoA is undertaking a regional surveillance initiative to determine POEs’ preparedness capacities to identify and assess TZD risks and mount adequate response and control measures.

There are no formal protocols or routine, integrated surveillance at Libyan POEs for any of the priority TZDs; NCDC and NCAH share information on zoonotic disease cases when requested, but these reports are not routinely communicated to POE officials. Health screenings for individuals crossing the border may be implemented following NCDC reports of an active outbreak of public health concern, such as HPAI; however, the same screening mechanism is not applied during atypical levels of seasonal influenza or brucellosis. As brucellosis is endemic in both countries, there is a lack of emphasis and concern for reporting cross-boundary spread from both the public and animal health sectors. Relevant surveillance information is shared with NCDC in Tripoli for analysis by the Surveillance Directorate. Outside of the required health checks and sampling of clinically ill/suspect animals, there is no routine TZD surveillance conducted by NCAH at POEs and/or quarantine locations; they rely on NCAH monitoring and sharing global epidemiology and outbreak reports.

### Detection

Individual travelers entering Tunisia are asked to present their personal identification documents; travel history and vaccination certificates may also be requested. If the individual appears ill, the patient can be isolated at the POE for further investigation. Strict sanitary and health certification, covering all five priority TZDs, are required ahead of animal importation. MoA/VS shares all requirements, such as testing for brucellosis and foot-and-mouth disease (FMD) two months prior to shipping, with importers/transporters and VS in countries of origin. Upon arrival animals are subjected to pre-approved quarantine centers, located off-site. MoA border officials review health certificates before regional VS who conduct exams, control checks and authorize release from quarantine. Tunisia does not currently export chicks/poultry or livestock; however, MoA is actively working to meet sanitary statuses for international trade standards to establish animal exports in the future. Domestic animals, such as dogs, cats, and birds are imported/exported with the required health certificates from VS.

In Libya, regulations, restrictions and screening for humans, animals, and goods at POEs are established in conjunction with the Customs Authority. Individual travelers are required to present their personal identification, travel history and vaccination certificates to IHMO border health officials. Health screenings are only conducted in response to national request, via NCDC Tripoli, during an active outbreak of public health concern in Libya or the surrounding region. In the event of a suspect case, patient information is collected and a sample is sent to NCDC Tripoli for processing. Health certificates and required documentation must be provided to and approved by NCAH border health officials prior to any animal (livestock and domestic pet) importation. Following verification of documents and a preliminary release by NCAH border officials, animals are transferred to the designated quarantine location off-site. There is no routine testing or clinical assessment performed on animals at the actual border crossing. Overall, NCAH relies heavily on health certificates to detect possible TZDs at importation. Libya and Tunisia have pre-existing contracts and health certificate requirements specifically for poultry export and import. Exported livestock receive health certificates, for the animal and source farm, issued by NCAH. They are inspected and spray acaricides are applied prior to movement. Livestock are prohibited from export and placed in quarantine if documentation is incomplete, or they appear sick/diseased. Following a predetermined time, they can be reassessed for export.

### Laboratory capacity

In Tunisia, samples collected from suspect/ill individuals at the POE are sent to the regional or national public health laboratory for diagnostic testing. MoPH relies on collaborations with the Pasteur Institute, which tests samples from both humans and animals, and the Charles Nicolle Hospital in Tunis for diagnostic and confirmation testing support for rabies and avian influenza. Test results are shared with national and border health MoPH officials to ensure proper surveillance, risk mitigation and control measures are implemented at the POE if necessary. Animal samples, including any observed ectoparasites collected by VS during quarantine are sent to the national animal health reference laboratory for testing, which can test for all priority TZDs. Laboratory results are communicated to national and border health MoA/VS officials. If suspect HPAI samples are confirmed positive, VS will immediately collect specimens from all quarantined poultry and coordinate with the country of origin.

Libya’s IHMO border health officials collect samples from any suspect/ill traveler and send samples to the NCDC national reference laboratory in Tripoli for testing, where all priority TZDs can be confirmed. Results are shared with both the Surveillance Department and IHMO, usually by phone. Depending on the causative agent, patients may be referred to a public hospital near the ground crossing for care. Samples collected from clinically ill animals in quarantine are sent to the NCAH national reference laboratory in Tripoli. Results are shared directly with NCAH and veterinarians at quarantine locations, as well as border health officials via WhatsApp or E-mail.

### Quarantine/Isolation

Symptomatic individuals are considered suspect cases upon entry into Tunisia, and when determined a transmission risk, are placed in isolation units available at all POEs. MoA/VS requires importers to identify a designated quarantine center prior to entering Tunisia. These off-site quarantine centers are privately owned and operated but must be approved by VS. Some countries are pre-approved for importation by VS based on their disease-free status for TZDs, aligning with WOAH recommendations. Once animals arrive at the quarantine centers, prophylactic vaccines for brucellosis and FMD are administered, regardless of health certificates, and Regional VS perform clinical examinations. Clinical animals are isolated and monitored for symptoms. If ectoparasites such as ticks are detected, samples are collected, animals are isolated and treated regardless of symptoms. The time an animal spends in quarantine depends on the disease risk and country of origin. Once quarantine has commenced, no new animals may be incorporated into the center. At the end of the prescribed quarantine period, animals are re-inspected and then approved for movement to their final destination by VS.

Libya’s international airports have established isolation units for ill travelers. Similar units were created at the Ras Ajdir and Dehiba Wazin border crossings during the COVID-19 pandemic and both contain an isolation clinic for patients. Similar to Tunisia, animal quarantine is conducted off-site. The quarantine locations for animals imported via ground crossings and seaports are administered by NCAH in coordination with Customs and Border Control. While there is no routine testing, VS are responsible for assessing, monitoring, and ensuring the health of animals at the quarantine location before they are moved to their final destination. If ectoparasites are present, acaricide treatment is provided. Sample collection and testing for vector-borne diseases is conducted only if the animal is clinically ill. The typical quarantine for animals is approximately 2 weeks but can be extended at the expense of the owner/transporter.

### Response

Any person who presents ill upon entry to Tunisia receives treatment in-country, regardless of nationality. Foreign patients are transported to and treated at regional public hospitals; Tunisian patients can be transferred to regional healthcare facilities or a university-level medical facility for care. Border health officials use Annex 2 of IHR as a decision tool and can notify any event of concern to the General Directorate of Health and IHR NFP. Rapid response teams (RRTs) with trained staff are available to support epidemiological investigations. MoPH leads case investigation in coordination with border health officials and the MoA/VS. In the case of an ill animal, the quarantine center informs MoA/VS, and Regional VS leads case investigation and response. While there are no designated RRTs, VS has standard operating procedures to support outbreak operations depending on the disease risk as well as surveillance and outbreak situational reports. Prior to confirmatory testing, MoA/VS implements response and control measures, including restriction of animal movement and isolation. Following the isolation tenure, animals are re-inspected for approval of movement. When an event is confirmed, there is continued coordination between the national-level MoPH and MoA/VS. VS will reiterate interdiction of all animal movement, notify the country of origin’s VS of the case, and negotiate on control measures, i.e. whether to cull the affected animals in country or return the animals.

If officials receive confirmation of HPAI from the originating flock or farm, the birds are culled onsite. Compensation for culled animals is only provided for cases of bovine tuberculosis. In addition, VS communicates and coordinates with other institutions in preparation for additional suspected cases at POEs.

If a traveler coming into Libya is suspected or confirmed to be ill, they are temporarily isolated, provided full care, and transferred to the nearest health facility with an isolation unit in their home country. If a traveler seeking medical consultation in Tunisia is suspected or confirmed at the POE, they are immediately transported by ambulance to a nearby public hospital with an isolation unit for treatment. NCDC’s Monitoring and Evaluation Department’s RRT is deployed from Tripoli to conduct an epidemiological investigation, as there are limited trained human resources at ground crossings. They will coordinate with NCDC Zoonotic Disease Control Unit, MOH, NCAH, National Food and Drug Administration (NFDA) and others, as necessary (and dependent on causative agent). Vector management and control for areas within POEs is a joint effort between municipal ESOs, NCDC, and the Civil Aviation Authority (when dealing with airports). In coordination with the Ministry of Interior, NCDC has the authority to establish and enforce isolation, quarantine or social distancing measures. For vector-borne concerns at POEs, NCDC would contact and coordinate with local-level ESOs, as well as the Pasteur Institute, to support vector control efforts. If there is any illness or suspicion of TZD infection in animals being imported or exported, local VS are contacted by NCAH to lead case investigation and manage response.While newly established NCAH RRTs are available in Tripoli to support case investigations at POEs, their resources and capabilities are limited. Any TZD suspect case at a POE is shared with NCDC and ESOs; laboratory confirmation is not necessary to initiate response actions. Suspected animals are quarantined and culled or returned to the country of origin, depending on the nature of the disease. Confirmed cases of brucellosis and/or CCHF require that animals are returned to the country of origin. Any detection of rabies, HPAI and/or RVF would result in animals being culled onsite.

### Communication and coordination

Human and animal border health officials coordinate on all priority TZDs in Tunisia. As noted above, both sectors are stationed at the same location within a POE so they can easily communicate and coordinate. All public health events that are detected at POEs are recorded and communicated to the national level. If an event is designated as a TZD, MoPH coordinates with MoA at the national level and involves Ministries of Interior, Trade, and Security, as necessary. Formal letters and informal WhatsApp messages are used to share information between the sectors. If a WHO or WOAH-notifiable disease such as HPAI or RVF is confirmed, the IHR NFP or Chief Veterinary Officer (CVO) notifies their respective international organization. If the case is confirmed in a foreign traveler, public health officials contact the IHR NFP of the patient’s home country.

Mechanisms for bidirectional communication exist in Libya at the subnational level; communication occurs mostly from POEs to the NCDC and onward to NCAH when a priority TZD is detected. While POEs house both NCDC and NCAH personnel, there is limited formal process for routine communication. Additionally, communication between NCDC and NCAH national offices in Tripoli can be delayed, as reporting systems and notification mechanisms differ. However, both NCDC and NCAH offices in Tripoli communicate with and report to WHO and WOAH, as necessary, through their respective IHR NFP and CVO.

Mechanisms exist for bilateral coordination between MoPH and NCDC (or through their respective IHR NFPs) for reports of HPAI, RVF and rabies. However, there is little outreach during cases of brucellosis and/or CCHF. While the COVID-19 pandemic required formalized, joint communications between NCDC and MoPH border health officials, post-pandemic there is no formal nor routine coordination mechanism for public health officials at ground crossings. It should be noted that there has been effective cross-border communication/coordination for human rabies cases. Libyan patients seeking care in Tunisia, driven by lack of available post-exposure prophylaxis (PEP), are identified by Tunisian border health officials who notify MoPH and they share outcomes with NCDC.

Animal health officials located at POEs communicate and coordinate on HPAI but there is little communication or coordination for other priority TZDs. In the past, NCAH and MoA/VS routinely shared case reports for brucellosis, RVF and rabies; however, this practice has been inconsistent for over ten years. Prior to this engagement, NCAH and MoA/VS were working together to strengthen avian influenza surveillance and create joint frameworks and guidance for all TZDs diseases that impact livestock and agriculture.

### Identified strengths and key recommendations for capacity building

The OHTAPZ systems map and corresponding narrative presented several key strengths and focus areas for TZD cross border operations for Tunisia and Libya. Identified strengths include transboundary disease educational and training materials for MoPH/MoA-VS and NCDC/NACH personnel that can be expanded to border health teams with minimal investment and time. In addition, MoA/VS and NCAH awareness campaigns for the general public and at-risk populations, such as herders and farmers, can be expanded to include public health for priority TZDs. Once in place, these trainings can be evaluated through tabletop and other simulated transboundary outbreak drills. There is routine communication between MoPH and NCDC at the national level and to their respective border health officials in reporting public health events and potential TZD risks in country and at POEs. In particular, there is strong bilateral coordination on HPAI, RVF and rabies. Both countries’ public and veterinary border health officials have mechanisms for surveillance, animal/livestock health certificates for import, patient isolation/quarantine, sample collection and laboratory confirmation. In many cases, Libya and Tunisia are capable of providing patient care, regardless of an individual’s nationality. However, while disease-specific coordination is in place, there is lack of formalized, disease-agnostic protocols for joint outbreak investigation between the two nations’ border health officials. Further, there is a lack of coordination on vector surveillance, response, and control operations at POEs.

The OHTAPZ experience established a mechanism for non-event communication and assessment between border health officials which can now be applied to the development of standard operating procedures (SOPs) for joint investigation, chain of custody, and response as well as the creation of integrated vector management measures at POEs. In addition, formalized national multisectoral One Health Committees could be established to support routine communication and coordination between countries. A One Health Cross-Border committee consisting of key focal points from national committees and POE operations should be established. The Cross-Border committee should have formalized terms of reference and regular meetings to share information, surveillance reports and ensure seamless application of joint SOPs. Finally, and potentially most critical, is the lack of a joint IHR-designated ground crossing POE [25]. As such, we recommend that Libyan and Tunisian officials prioritize the joint designation of at least one border crossing, preferably Ras Ajdir. The results of this pilot process can support priority actions for capacity building that will build towards targeted capacities under IHR monitoring and evaluation frameworks [26]. Table 4 provides a detailed summary of the joint capacity gaps and targeted actions identified to strengthen bilateral capabilities. The provided recommendations can be incorporated into a strategic roadmap to target specific initiatives, based on priority and funding, for advocacy and implementation.

**Table 4:** OHTAPZ joint capacity findings and recommendations

Capacity	Joint Findings	Recommendations
Prevention	Lack of training on TZD disease detection for POE officials/border health officers	Incorporate existing TZD awareness raising and educational trainings provided by MoPH/MoA-VS and NCDC/NCAH to border health officials. Once in place, establish regular/routine joint training opportunities. These trainings can be evaluated through tabletop and other simulated transboundary outbreak drills
Prevention, Surveillance & Response	Lack of coordination on vector surveillance, response, and control at border crossings	Implement integrated vector management measures at POEs from prevention to surveillance and response which can be piloted at one border crossing first before expanding to other POE settings. Incorporate national-level sectors that oversee or are developing vector control programs
Response	No protocol for joint outbreak (animal or human) investigation between POE officials in Libya and Tunisia	Apply OHTAPZ methodology and cross-border network to complete a mapping of critical One Health stakeholders at POEs to develop of standard operating procedures (SOPs) for joint investigation, chain of custody, and response
Communication & Coordination	Lack of formalized One Health structures to support ministerial- and bilateral level communication and coordination for priority TZDs	Define, formalize and test MoPH-NCDC, MoA/VS-NCAH, POE-POE, and bilateral communication channels for surveillance of and detection and response to priority TZDs. National multisectoral One Health Committees (OHCs) should be established to support routine communication, coordination within countries In addition, a One Health Cross-Border committee consisting of key focal points from national OHCs and POE operations should be established. The Cross-Border committee should have formalized terms of reference and regular meetings to share information, surveillance reports and ensure seamless application of joint SOPs
Other	No jointly designated ground crossing POE	Designate one land border crossing, preferably Ras Ajdir, for joint implementation of IHR and expand current assessment of existing capacities using IHR POE assessment tool

The table summarizes the joint findings from the OHTAPZ methodology categorized based on the seven competencies (prevention; surveillance; detection; laboratory capacity; quarantine/isolation; response; and communication and coordination) with specific recommendations for capacity building in cross border operations. Only competencies where key gaps were identified are captured in the table

## Discussion

The OHTAPZ methodology initiates discussions between crucial multisectoral actors responsible for transboundary disease operations at POEs, provides a comprehensive overview of national and transboundary operations for priority TZDs, and identifies existing structures and networks that can be strategically leveraged for other disease threats or targeted for future health strengthening initiatives. These findings serve to benefit national, bilateral and regional efforts to improve and sustain prevention and management of priority TZDs.

### Multilateral TZD prioritization

Transboundary disease prioritization is a critical step in focusing limited resources for preparedness, detection, and response and optimizing operational planning efforts. TZDs whether endemic, emerging and/or re-emerging, necessitate multisectoral and regional coordination and cooperation, superseding competing priorities. Reaching joint consensus between human, animal, environmental, border health, security and other relevant sectors is essential. Using a selection process and qualifying criteria for disease prioritization elicits interest and priorities between sectors/countries and contributes to bridging potential differences. For cross-border threats such as TZDs and TADs, a narrowed list of mutually agreed upon priority diseases encourages cooperative and complementary approaches to prevention, detection, surveillance, and response both within and between nations or regional partners [27, 28]. We recognize that our pilot process was limited in its One Health scope as there was no direct involvement from environmental health, security, or customs and border sectors due to programmatic constraints. While equities were partially captured by public health, veterinary and border colleagues, future iterations will formalize and expand stakeholder participation. Expanding stakeholders in an effort to balance representation may yield different outcomes during prioritization based on knowledge and firsthand experience, alleviate subjectivity, enhance transparency, and help join the natural and social sciences [28, 29]. As the One Health approach continues to be embraced and implemented, countries and regions must make concerted efforts to expand involvement beyond the human, animal and environmental health sectors, incorporate non-traditional ministries and actors, and consider alternatives to top-down decision-making. Ultimately, in order to build strong and sustainable solutions, stakeholders must be engaged and committed to applying the priority disease list and implementing effective mitigation strategies at the national and transboundary levels.

### Joint designation of ground crossings

Ground crossings have unique considerations compared to other POEs. These border locations serve as conduits for commercial and commuter travel; experience different modes and numbers of transportation, such as trains, trucks, lorries, automobiles, buses, bicycles, or animals traveling on foot; and have diverse infrastructure and resource availability, which includes staffing, electricity, cell phone service, etc. [30]. Most ground crossings around the world are unofficial or informal; however, the lack of IHR-designation does not negate the critical need for health security infrastructure and implementation. Moreover, these diverse POE settings are critical weak points and challenging environments for consistent and sustainable IHR capacity building and thus prevention, detection and control of TZDs [30].

The IHR calls on States Parties to designate specific POEs within their borders to help prioritize capacity building effort for compliance. Notably, the IHR only “suggests” the inclusion of ground crossings for designation “where justified for public health reasons” [3, 28]. WHO recommends that countries review and assess known public health risks that could be encountered along travel routes for cargo and travelers prior to arrival, epidemiological status in and around the POE locale, and facilities and logistical capabilities of POEs [26, 30]. Cooperation between neighboring countries for joint designation and/or bilateral agreements towards disease prevention or control is “encouraged” rather than mandated [25, 30]. Nevertheless, if cooperation and/or agreements exist between countries, there is potential for joint designation of one or more POEs, which would encourage bilateral communication and broader capacity building [26]. The joint designation of a ground crossing between Libya and Tunisia could serve as a symbolic first step in bilateral commitment to strengthening priority TZDs capacities at POEs.

### Elevating priority areas for TZD capacity building

As a result of our systems mapping, we identified evident gaps in transboundary communication and coordination for vector-borne diseases at both the ministerial/subnational and border levels. While RVF and CCHF are considered emerging in Libya and Tunisia, their expansion and amplification, like other vector-borne diseases, is accelerated by environmental change and globalization, and intrinsically connected to ecological, political and socioeconomic drivers [17]. In addition to the political instability thwarting routine control efforts in Libya, porous borders and unofficial crossing of humans, animals, and wildlife challenge national and bilateral disease detection and management. POEs serve as an ideal location for cross-border case detection and notification, coordination on vector control and management initiatives, as well as routine surveillance collaboration. Following results from this OHTAPZ pilot, Libyan NCDC officials were motivated to establish a new unit within the Zoonotic Disease Control Directorate (Tripoli) specifically responsible for vector collection, identification, and implementation of control measures at POEs. At present, the proposed unit has been approved but operations have not yet been implemented. In addition, NCDC is developing a vector monitoring and control curriculum for border health officials to be piloted at airports before expanding to other POEs. This re-approach and initiation of targeted vector control activities at POEs is just one example of how OHTAPZ can assist in identifying and elevating priority areas for TZD capacity building.

## Conclusions

POEs serve as ideal settings for building enhanced preparedness, prevention, detection, notification, response, and coordination mechanisms for health systems. The OHTAPZ methodology supported a pilot assessment and analysis of existing networks, policies and operations for the prevention, detection, and response to TZD threats at formal ground crossings between Libya and Tunisia. This first application of OHTAPZ in a bilateral setting provides an opportunity for review, revision and application in additional cross-border settings incorporating lessons learned such as expanded stakeholder engagement; interactive tabletop and simulation exercises aligned with WHO and WOAH competencies; and TZD self-assessment tools for POEs to encourage routine monitoring and evaluation. Overall, OHTAPZ serves as a useful methodology in developing One Health capacity at ground crossings and supports multisectoral preparedness and response operations at POEs for TZDs.

## Data Availability

All data generated or analyzed during this study are included in this published article.

## Abbreviations

CCHF: Crimean-Congo hemorrhagic fever
COVID-19: Coronavirus disease 2019
EIDSS: Electronic integrated disease surveillance system
ESO: Environmental sanitation office, Libya
FAO: Food and agriculture organization
FMD: Foot-and-mouth disease
GF-TAD: Global framework for the progressive control of transboundary animal diseases
HPAI: Highly-pathogenic avian influenza
IHMO: International health monitoring office, Libya
IHR: International health regulations
JEE: Joint external evaluation
MoA/VS: ministry of agriculture, water resources and fisheries/ general directorate for veterinary services, Tunisia
MOH: Ministry of health
MOI: Ministry of interior
MoPH: Ministry of public health, Tunisia
NCAH: National centre for animal health, Libya
NCDC: National centre for disease control, Libya
NFDA: National food and drug administration, Libya
NFP: National focal point
OHSAPZ: One health systems assessment for priority zoonoses
OHTAPZ: One health transboundary assessment for priority zoonoses
POE: Point of entry
ProMed: Programing for monitoring emerging diseases
RRT: Rapid response team
RVF: Rift Valley fever
SARS: Severe acute respiratory syndrome
SOP: Standard operating procedure
SPAR: States party self-assessment annual report
TAD: Transboundary animal disease
TZD: Transboundary zoonotic disease
VS: Veterinary services
WAHIS: World animal health information system
WHO: World health organization
WOAH: World organisation for animal health
